# Administration of All-Trans Retinoic Acid to Pregnant Sows Alters Gut Bacterial Community of Neonatal Piglets With Different Hoxa1 Genotypes

**DOI:** 10.3389/fmicb.2021.712212

**Published:** 2021-07-26

**Authors:** Haimei Zhou, Huadong Wu, Yixin Chen, Wanjie Zou, Wei Lu, Yuyong He

**Affiliations:** ^1^Jiangxi Province Key Laboratory of Animal Nutrition/Engineering Research Center of Feed Development, Jiangxi Agricultural University, Nanchang, China; ^2^Department of Animal Science, Jiangxi Agricultural Engineering College, Zhangshu, China; ^3^College of Animal Science and Technology, Jiangxi Agricultural University, Nanchang, China

**Keywords:** Hoxa1 mutation, all-trans retinoic acid, pregnant sows, gut bacterial community, neonatal piglets

## Abstract

Administration of all-trans retinoic acid (ATRA) to pregnant sows improves developmental defects of Hoxa1^–/–^ fetal pigs, and this study aimed to explore the influence of maternal ATRA administration during pregnancy on gut microbiota of neonatal piglets. Samples of jejunal and ileal meconium of neonatal piglets before suckling were collected including 5 Hoxa1^–/–^ and 20 non-Hoxa1^–/–^ (Hoxa1^+/+^ and Hoxa1^+/−^) neonatal piglets from the control group and 5 Hoxa1^–/–^ and 7 non-Hoxa1^–/–^ neonatal piglets from the experimental group. Results indicated that Hoxa1 mutation shaped the bacterial composition of the jejunum and ileum of neonatal piglets and Hoxa1^–/–^ neonatal piglets had significantly higher diversity and species richness, higher relative abundance of phylum Bacteroidetes, lower relative abundances of phylum Firmicutes and genus *Lactobacillus*, and lower ratio of Firmicutes to Bacteroidetes than non-Hoxa1^–/–^ neonatal piglets. After maternal ATRA administration, Hoxa1^–/–^ neonatal piglets had significantly higher diversity and species richness, higher relative abundances of two bacterial phyla (Bacteroidetes and Proteobacteria), and lower relative abundances of phylum Firmicutes and genus *Lactobacillus* in the jejunum than non-Hoxa1^–/–^ neonatal piglets. Hoxa1^–/–^ neonatal piglets delivered by sows with maternal ATRA administration had lower diversity and species richness and higher relative abundance of phylum Firmicutes in the jejunum than Hoxa1^–/–^ neonatal piglets born by sows with no maternal ATRA administration. Non-Hoxa1^–/–^ neonatal piglets delivered by sows with maternal ATRA administration had higher diversity and species richness and significantly lower relative abundances of phyla Firmicutes and Actinobacteria and genus *Lactobacillus* in the ileum than non-Hoxa1^–/–^ neonatal piglets born by sows with no maternal ATRA administration. Hoxa1 mutation decreased the expression of bacterial genes involved in ABC transporters, purine metabolism, and aminoacyl-tRNA biosynthesis and increased the expression of bacterial genes involved in two-component system, starch and sucrose metabolism, and arginine and proline metabolism. Maternal ATRA administration decreased the expression of bacterial genes involved in arginine and proline metabolism, peptidoglycan biosynthesis, and fatty acid biosynthesis. Hoxa1 mutation resulted in bacterial dysbiosis of the small intestine of Hoaxa1^–/–^ neonatal piglets, and maternal ATRA administration restored the bacterial dysbiosis of Hoxa1^–/–^ neonatal piglets and altered the bacterial composition of the small intestine of non-Hoxa1^–/–^ neonatal piglets.

## Introduction

Gut microbiota is integral to feed digestion, nutrient absorption and metabolism, immune response, and gastrointestinal development (Morgavi et al., 2015), and the colonization of intestinal microbiota during early life could further influence the subsequent microbiota of adult host (Ben Salem et al., 2005). Many studies demonstrated that the intestine of prenatal animals really has microorganism (Alipour et al., 2018; Stinson et al., 2019; Hummel et al., 2020; Bi et al., 2021; Husso et al., 2021), and at present, no literature on the differences in intestinal microbiota composition between mutant and wild-type fetuses is found, but for postnatal individuals, there are differences in gut microbiota between mutant and wild-type host, for example, nucleotide-binding oligomerization domain-containing protein 2 (NOD2) mutation caused Crohn’s disease (CD) (Hampe et al., 2001; Ogura et al., 2001) and Crohn’s disease individuals had lower bacterial diversity than healthy controls (Joossens et al., 2011). Cystic fibrosis transmembrane conductance regulator (CFTR) mutation resulted in multiorgan defects, and CFTR^–/–^ mice had significantly lower alpha diversity of intestinal bacterial community (*p* < 0.05) and had reduced relative abundance of protective species such as *Acinetobacter lwoffii* and Lactobacilliales members compared with wild-type mice (Lynch et al., 2013). Methyl-CpG-binding protein 2 (MeCP2) mutation developed into the Rett syndrome (RTT), and RTT patients had significantly less diversity in gut bacteria community compared with healthy controls (*p* < 0.01). Meanwhile, RTT individuals had the most abundant phylum of Actinobacteria, but healthy controls had the most abundant phylum of Firmicutes, and a significant decrease of Bacteroidetes was observed in RTT subjects (Strati et al., 2016).

Gene mutation not only can alter intestinal microbiota composition of postnatal animals but also can cause abnormal phenotypes of fetuses: cytochrome C oxidase subunit IV isoform 1 (COX4I1) mutation caused short stature and poor weight gain (Abu-Libdeh et al., 2017); mutations of Huntington (HTT) exhibited fetal ear defects (Murthy et al., 2019); K^+^ channels Kir4.1 (KCNJ10) gene mutation resulted in seizures and ataxia (Ai Dhaibani et al., 2018); gamma-1 adaptin gene (*Ap1g1*) mutation developed abnormalities of the inner ear and testes (Johnson et al., 2016); WD repeat domain phosphoinositide-interacting protein 2 (WIPI2) mutation led to skeletal and cardiac abnormalities (Jelani et al., 2019); fibroblast growth factor receptor 2 (FGFR2) mutation induced midfacial hypoplasia and bilateral syndactyly of the hands and feet (Giancotti et al., 2014); solute carrier family 26a member 4 (SLC26A4) mutation developed deafness (Nonose et al., 2018); a Q186K mutation in Hoxa2 resulted in external ear malformation (Alasti et al., 2008); and the Hoxa1 mutation of g.50111251 G > TC developed abnormal auricle and external auditory canal, dyspnea, and even death in newborn piglets (Qiao et al., 2015). Some defects of phenotypes can be rescued by feeding special chemicals to pregnant animals during pregnancy at a specific time: administration of exogenous RA to pregnant mice at a dose of 2.5 mg/kg on day embryonic 7.5 or embryonic 8.5 effectively repaired the Hoxa1 mutant mice from inner ear defects (Pasqualetti et al., 2001), and all-trans retinoic acid (ATRA) administration to pregnant sows at the level of 4 mg/kg body weight on 14 days postcoitum (dpc) was also effective for the repair of ear defects of Hoxa1^–/–^ fetal pigs (Zhou et al., 2021). As mentioned above, the abnormal phenotypes of fetus caused by gene mutation can be rescued *via* chemical administration during gestation, but there is no information if maternal administration of chemicals can also change the intestinal bacterial composition of mutant and wild-type individuals. Understanding the influence of maternal administration with special chemicals during pregnancy on the community composition and function of the neonatal gut bacteria may help to develop strategies to prevent young animals from suffering from some diseases and to guide the healthy development of the offspring. Our previous studies demonstrated that maternal administration with ATRA at the level of 4 mg/kg body weight on 14 dpc had the best effects in repairing ear defects of Hoxa1^–/–^ fetal pigs, and the aims of this study are (1) to find out if maternal administration with ATRA can alter the intestinal bacterial compositions of Hoxa1^–/–^ and non-Hoxa1^–/–^ fetal piglets and (2) to compare the differences in intestinal bacterial compositions of neonate piglets between Hoxa1^–/–^ and non-Hoxa1^–/–^ genotypes.

## Materials and Methods

### Animals and Sample Collection

Eight Hoxa1^+/–^ sows derived from one Chinese Erhualian founder boar and one Shaziling founder sow were mated to one healthy Hoxa1^+/–^ boar and randomly assigned to a control group (six sows) and an experimental group (two sows). Pregnant sows in the control group were orally administered with ATRA at a level of 0 mg/kg body weight, and pregnant sows in the experimental group were orally administered with ATRA at a level of 4 mg/kg body weight (Zhou et al., 2021).

After birth, all samples were collected before suckling. Samples of ears of all neonatal piglets were collected and stored in EP tubes containing 75% alcohol for Hoxa1 genotyping. Hoxa1^–/–^ piglets delivered by sows from the control group had ear defects, and the ear defects of Hoxa1^–/–^ piglets born by sows from the experimental group were effectively repaired; all non-Hoxa1^–/–^ (Hoxa1^+/+^, Hoxa1^+/–^) either from the control group or the experimental group had normal ears (Zhou et al., 2021). Samples of meconium of all neonatal piglets were collected from jejunal and ileal sections by exsanguination after anesthetization with pentobarbital sodium (100 mg/kg body weight) according to the protocol approved by the Animal Ethics Committee of Jiangxi Agricultural University and immediately stored at −80°C for microbiome analysis. A total of 37 neonatal piglets were sampled, namely, 25 piglets (5 Hoxa1^–/–^ and 20 non-Hoxa1^–/–^) from the control group and 12 piglets (5 Hoxa1^–/–^ and 7 non-Hoxa1^–/–^) from the experimental group.

### DNA Extraction, Amplification, and Sequencing

Genomic DNA of intestinal meconium was extracted with the QIAamp DNA Stool Mini Kit (Qiagen, Hilden, Germany) according to the instructions of the manufacturer, and the quantity and quality of DNA were measured with NanoDrop^TM^ 2000/2000c Spectrophotometer (Thermo Fisher Scientific, Waltham, MA, United States). The V3–V4 region of bacterial 16S rRNA genes was amplified using primers 341F (5′-CCTACGGGNGGCWGCAG-3′) and 806R (5′-GGACTACHVGGGTATCTAAT-3′). PCR reactions were performed in triplicate 50 μl mixture containing 5 μl of 10 × KOD buffer, 5 μl of 2 mM dNTPs, 3 μl of 25 mM MgSO_4_, 1.5 μl of each primer (10 μM), 1 μl of KOD polymerase, and 100 ng of template DNA. The PCR conditions consisted of initial denaturation at 94°C for 2 min, followed by 30 cycles of denaturation at 98°C for 10 s, annealing at 65°C for 30 s, elongation at 768°C for 30 s, and finally 68°C for 5 min. The PCR products were subsequently subjected to electrophoresis on 2% agarose gel and stained with ethidium bromide, and the targeted fragment size was purified using the AMPure XP Beads (Beckman Agencourt, Brea, CA, United States) according to the manufacturer’s instructions and quantified using ABI StepOnePlus Real-Time PCR System (Life Technologies, Foster City, CA, United States). Purified amplicons were pooled in equimolar and paired-end sequenced (PE250) on an Illumina HiSeq 2500 platform (Illumina, San Diego, CA, United States) according to standard protocols. The raw reads were deposited into the NCBI Sequence Read Archive (SRA) database (accession number: SRP239498).

### Sequence Processing and Data Statistical Analysis

Raw reads were further filtered using FASTP (version 0.18.0) (Chen et al., 2018), and paired end clean reads were merged as raw tags using FLASH (version 1.2.11) (Magoč and Salzberg, 2011) with a minimum overlap of 10 bp and mismatch error rates of 20%. The clean tags were clustered into operational taxonomic units (OTUs) at 97% sequence similarity using UPARSE (version 9.2.64) pipeline (Edgar, 2013). According to the algorithm principle, the sequences with the highest occurrence frequency were selected as the representative sequence of OTUs. The representative OTU sequences were classified into organisms by a naive Bayesian model using RDP classifier (version 2.2) (Wang et al., 2007) based on the SILVA database (version 132) (Pruesse et al., 2007) with a confidence threshold value of 0.8 to obtain taxonomic information and the community composition of each sample at various classification levels; the abundance statistics of each taxonomy was visualized using Krona (version 2.6) (Ondov et al., 2011).

Chao1 and Shannon index were calculated in QIIME (version 1.9.1) (Caporaso et al., 2010) and alpha index comparison between groups was calculated by Welch’s *t*-test in R project Vegan package (version 2.5.3) (Oksanen et al., 2010). Bacterial community structure and composition were compared using non-metric multidimensional scaling (NMDS) analysis by means of weighed UniFrac distances in R using the metaMDS function (Caporaso et al., 2012). Permutational multivariate analyses of variance (PERMANOVA; “adonis and anosim” in vegan R package) with 999 random permutations were performed to assess the influence of substrate on the community variances. The functional potentials of intestinal bacteria were predicted using Tax4Fun package in R software based on the Kyoto Encyclopedia of Genes and Genomes (KEGG) Orthology (KO) terms at level 3 with the observed 16S rRNA gene sequences (Aßhauer et al., 2015; Kanehisa et al., 2015). Analysis of function difference between groups was calculated by Welch’s *t*-test in R project Vegan package (version 2.5.3) (Oksanen et al., 2010).

## Results

### Bacterial Richness and Alpha Diversity

Table 1 shows the alteration of richness and diversity of small intestinal bacterial community between Hoxa1^–/–^ and non-Hoxa1^–/–^ neonatal piglets within the same treatment group. Hoxa1^–/–^ neonatal piglets either from the control group or the experimental group had significantly higher OTU (*p* < 0.01), Chao1 (*p* < 0.01), and Shannon (*p* < 0.05) of jejunal bacterial community than non-Hoxa1^–/–^ neonatal piglets, respectively. In the control group, Hoxa1^–/–^ neonatal piglets had significantly higher OTU (*p* < 0.05), Chao1 (*p* < 0.05), and Shannon (*p* < 0.01) of ileal bacterial community than non-Hoxa1^–/–^ neonatal piglets. However, in the experimental group, Hoxa1^–/–^ neonatal piglets had no significantly higher OTU, Chao1, and Shannon of ileal bacterial community than non-Hoxa1^–/–^ neonatal piglets, respectively (*p* > 0.05).

**TABLE 1:** Diversity comparison between Hoxa1^–/–^ and Non-Hoxa1^–/–^ newly born piglets in the same treatment group.

	Control group			Experimental group		
		
	Hoxa1^–/–^ piglets	Non-Hoxa1^–/–^ piglets	*p*-value	Hoxa1^–/–^ piglets	Non-Hoxa1^–/–^ piglets	*p*-value
Diversity of jejunal bacteria	KD-1	KD-2		KC-1	KC-2	
OTU	538.83 ± 75.02	180.67 ± 12.02	0.005	449.67 ± 24.59	309.67 ± 18.97	0.001
Chao1	806.80 ± 82.12	257.78 ± 14.97	0.001	721.33 ± 17.53	526.87 ± 40.38	0.003
Shannon	5.37 ± 0.68	3.43 ± 0.34	0.036	4.65 ± 0.27	3.07 ± 0.24	0.011
Diversity of ileal bacteria	HD-1	HD-2		HC-1	HC-2	
OTU	622.00 ± 67.02	203.33 ± 14.39	0.024	358.50 ± 44.77	351.00 ± 87.78	0.456
Chao1	659.05 ± 99.12	287.37 ± 23.32	0.030	573.82 ± 60.88	479.58 ± 78.72	0.604
Shannon	6.51 ± 0.82	3.20 ± 0.12	0.009	4.35 ± 0.35	3.67 ± 0.46	0.842

Table 2 indicates the change of richness and diversity of intestinal bacterial community of neonatal piglets with the same genotype between the control group and the experimental group. Hoxa1^–/–^ neonatal piglets from the control group had no significantly higher OTU, Chao1, and Shannon of jejunal and ileal bacterial community than Hoxa1^–/–^ neonatal piglets from the experimental group, respectively (*p* > 0.05). Non-Hoxa1^–/–^ neonatal piglets from the control group had significantly lower OTU (*p* < 0.01) and Chao1 (*p* < 0.01) but had no significantly higher Shannon (*p* > 0.05) of jejunal bacterial community than non-Hoxa1^–/–^ neonatal piglets from the experimental group, respectively. Non-Hoxa1^–/–^ neonatal piglets from the control group had no significant lower OTU, Chao1, and Shannon of ileal bacterial community than that of non-Hoxa1^–/–^ neonatal piglets from the experimental group (*p* > 0.05).

**TABLE 2:** Diversity comparison of newly born piglets with the same genotype between the control group and the experimental group.

	Hoxa1^–/–^ piglets			Non-Hoxa1^–/–^ piglets		
		
	Control group	Experimental group	*p*-value	Control group	Experimental group	*p*-value
Diversity of jejunal bacteria	KD-1	KC-1		KD-2	KC-2	
OTU	538.83 ± 75.02	449.67 ± 24.59	0.301	180.67 ± 12.02	309.67 ± 18.97	0.000
Chao1	806.80 ± 82.12	721.33 ± 17.53	0.352	257.78 ± 14.97	526.87 ± 40.38	0.001
Shannon	5.37 ± 0.68	4.65 ± 0.27	0.357	3.43 ± 0.34	3.07 ± 0.24	0.407
Diversity of ileal bacteria	HD-1	HC-1		HD-2	HC-2	
OTU	622.00 ± 67.02	358.50 ± 44.77	0.064	203.33 ± 14.39	351.00 ± 87.78	0.123
Chao1	659.05 ± 99.12	573.82 ± 60.88	0.124	287.37 ± 23.32	479.58 ± 111.32	0.140
Shannon	6.51 ± 0.82	4.35 ± 0.35	0.056	3.20 ± 0.12	3.67 ± 0.46	0.362

These results demonstrate that the g.50111251 G > TC mutation in Hoxa1 significantly increased the OTU, Chao1, and Shannon of jejunal and ileal bacterial community, respectively, when comparing Hoxa1^–/–^ neonatal piglets with non-Hoxa1^–/–^ neonatal piglets. Maternal ATRA administration decreased the OTU, Chao1, and Shannon of jejunal and ileal bacterial community of Hoxa1^–/–^ neonatal piglets but increased the OTU, Chao1, and Shannon of jejunal and ileal bacterial community of non-Hoxa1^–/–^ neonatal piglets with an exception of Shannon in ileal bacteria.

### Bacterial Community Composition

**FIGURE 1:**
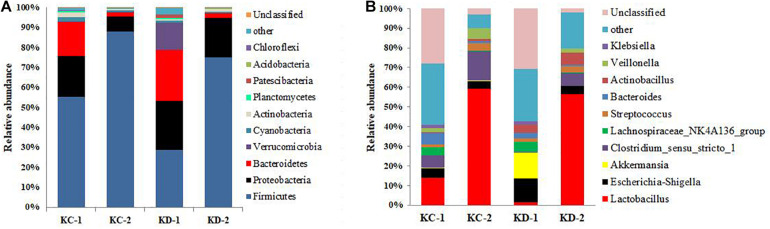
Bacterial community composition of jejunal digesta. **(A)** Phylum level; **(B)** genus level. KC-1: bacterial composition of jejunal digesta sampled from Hoxa1^− /−^ neonatal piglets delivered by sows administered with ATRA, KC-2: bacterial composition of jejunal digesta sampled from non-Hoxa1^− /−^ neonatal piglets delivered by sows administered with ATRA, KD-1: bacterial composition of jejunal digesta sampled from Hoxa1^− /−^ neonatal piglets delivered by sows administered without ATRA, KD-2: bacterial composition of jejunal digesta sampled from non-Hoxa1^− /−^ neonatal piglets delivered by sows administered without ATRA.

**FIGURE 2:**
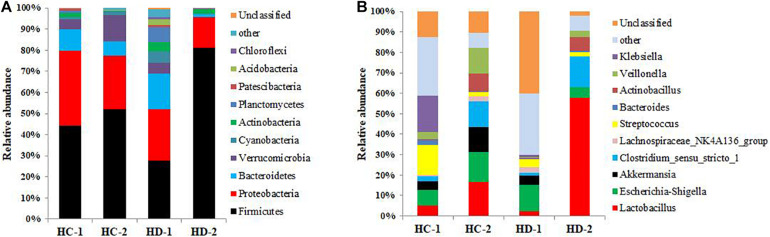
Bacterial community composition of ileal digesta. **(A)** Phylum level; **(B)** genus level. HC-1: bacterial composition of ileal digesta sampled from Hoxa1^− /−^ neonatal piglets delivered by sows administered with ATRA, HC-2: bacterial composition of ileal digesta sampled from non-Hoxa1^− /−^ neonatal piglets delivered by sows administered with ATRA, HD-1: bacterial composition of ileal digesta sampled from Hoxa1^− /−^ neonatal piglets delivered by sows administered without ATRA, HD-2: bacterial composition of ileal digesta sampled from non-Hoxa1^− /−^ neonatal piglets delivered by sows administered without ATRA.

The compositions of bacteria in the jejunal and ileal meconium are presented in Figures 1, 2. The phyla Firmicutes and Proteobacteria were the dominant bacteria in both meconium, and Hoxa1 mutation altered the relative abundances of intestinal bacteria at the phylum level (Figures 1A, 2A). In the control group, Hoxa1^–/–^ neonatal piglets had lower abundance of Firmicutes and higher abundances of Proteobacteria, Bacteroidetes, and Verrucomicrobia than non-Hoxa1^–/–^ neonatal piglets in jejunal (KD-1:KD-2) and ileal meconium (HD-1:HD-2), respectively. In the experimental group, Hoxa1^–/–^ neonatal piglets still had lower abundance of Firmicutes and higher abundances of Proteobacteria and Bacteroidetes than non-Hoxa1^–/–^ neonatal piglets in jejunal (KC-1:KC-2) and ileal (HC-1:HC-2) meconium, respectively, but Hoxa1^–/–^ neonatal piglets from the experimental group had higher abundance of Firmicutes and lower abundances of Bacteroidetes and Verrucomicrobia than Hoxa1^–/–^ neonatal piglets from the control group in jejunal (KC-1:KD-1) and ileal meconium (HC-1:HD-1), respectively. Maternal administration with ATRA also had an influence on the phyla bacteria abundance of non-Hoxa1^–/–^ neonatal piglets, and the data in Figures 1A, 2A indicated that non-Hoxa1^–/–^ neonatal piglets from the experimental group had higher abundance of Firmicutes and lower abundances of Proteobacteria and Bacteroidetes than non-Hoxa1^–/–^ neonatal piglets from the control group in the jejunal meconium (KC-2:KD-2), but had lower abundance of Firmicutes and higher abundances of Proteobacteria, Bacteroidetes, and Verrucomicrobia than non-Hoxa1^–/–^ neonatal piglets from the control group in the ileal meconium (HC-2:HD-2).

At the genus level, data in Figure 1B showed that in the control group, Hoxa1^–/–^ neonatal piglets had lower abundances of *Lactobacillus*, *Actinobacillus*, and *Streptococcus* and higher abundances of *Escherichia–Shigella*, *Akkermansia*, and *Lachnospiraceae_NK4A136_group* than non-Hoxa1^–/–^ neonatal piglets, respectively, in the jenunal meconium (KD-1:KD-2), but in the experimental group, Hoxa1^–/–^ neonatal piglets had lower abundances of *Lactobacillus*, *Clostridium_sensu_stricto_1*, and *Veillonella* and higher abundances of *Escherichia–Shigella*, *Bacteroides*, and *Lachnospiraceae_NK4A136_group* than non-Hoxa1^–/–^ neonatal piglets, respectively, in the jejunal meconium (KC-1:KC-2). Hoxa1^–/–^ neonatal piglets from the experimental group had higher abundances of *Lactobacillus*, *Clostridium_sensu_stricto_1*, and *Bacteroides* and lower abundances of *Escherichia–Shigella*, *Lachnospiraceae_NK4A136_group*, and *Akkermansia* than Hoxa1^–/–^ neonatal piglets from the control group, respectively, in the jejunal meconium (KC-1:KD-1). Results in Figure 2B indicated that in the control group, Hoxa1^–/–^ neonatal piglets had lower abundances of *Lactobacillus*, *Clostridium_sensu_stricto_1*, *Bacteroides*, *Actinobacillus*, and *Veillonella* and higher abundances of *Escherichia–Shigella*, *Akkermansia*, *Streptococcus*, and *Lachnospiraceae_NK4A136_group* than non-Hoxa1^–/–^ neonatal piglets, respectively, in the ileal meconium (HD-1:HD-2), but in the experimental group, Hoxa1^–/–^ neonatal piglets had lower abundances of *Lactobacillus*, *Escherichia–Shigella*, *Akkermansia*, and *Clostridium_sensu_stricto_1* and higher abundances of *Streptococcus* and *Klebsiella* than non-Hoxa1^–/–^ neonatal piglets, respectively, in the ileal meconium (HC-1:HC-2). Hoxa1^–/–^ neonatal piglets from the experimental group had higher abundances of *Lactobacillus*, *Clostridium_sensu_stricto_1*, *Streptococcus*, *Bacteroides*, *Veillonella*, and *Klebsiella* and lower abundances of *Escherichia–Shigella* and *Akkermansia* than Hoxa1^–/–^ neonatal piglets from the control group, respectively, in the ileal digesta (HC-1:HD-1).

### Relative Abundance of Differentially Jejunal Bacterial Community

The relative abundances of differential bacterial taxa in the jejunal meconium (at least one of the relative abundances is greater than 0.01%) are presented as percentage in Tables 3, 4. The results in Table 3 indicate that four bacterial taxa with differential abundances at the phylum level and 10 bacterial taxa with differential abundances at the genus level were identified in the samples of jejunal meconium between Hoxa1^–/–^ and non-Hoxa1^–/–^ piglets from the control group, and Hoxa1^–/–^ neonatal piglets had significantly lower relative abundances of Firmicutes (*p* < 0.01) at the phylum level and of *Lactobacillus* (*p* < 0.01), *Staphylococcus* (*p* < 0.01), and *Veillonella* (*p* < 0.01) at the genus level, respectively, in the jejunal digesta than non-Hoxa1^–/–^ neonatal piglets. After maternal ATRA administration, Hoxa1^–/–^ neonatal piglets from the experimental group still had significantly lower relative abundances of Firmicutes (*p* < 0.01) at the phylum level and of *Lactobacillus* (*p* < 0.01) at the genus level, respectively, in the jejunal digesta than non-Hoxa1^–/–^ neonatal piglets.

**TABLE 3:** The differential jejunal bacterial community of newborn piglets between different genotypes within the same treatment group (%).

	Control group				Experimental group		
			
	Hoxa1^–/–^ piglets (KD-1)	Non-Hoxa1^–/–^ piglets (KD-2)	*p*-value		Hoxa1^–/–^ piglets (KC-1)	Non-Hoxa1^–/–^ piglets (KC-2)	*p*-value
**Phylum level**				**Phylum level**			
Firmicutes	28.78 ± 7.11	75.09 ± 9.32	0.003	Firmicutes	55.36 ± 8.11	88.09 ± 4.68	0.008
Bacteroidetes	25.47 ± 5.54	2.54 ± 1.11	0.008	Proteobacteria	20.18 ± 3.06	7.28 ± 3.01	0.013
Patescibacteria	1.63 ± 0.62	0.01 ± 0.00	0.046	Bacteroidetes	17.05 ± 5.09	2.16 ± 0.19	0.032
Planctomycetes	0.56 ± 0.20	0.00 ± 0.00	0.039				
**Genus level**				**Genus level**			
*Lactobacillus*	1.39 ± 0.47	56.63 ± 10.10	0.003	*Lactobacillus*	14.02 ± 8.01	59.32 ± 7.68	0.002
*Alloprevotella*	1.18 ± 0.45	0.00 ± 0.00	0.046	*Acinetobacter*	0.71 ± 0.18	0.04 ± 0.01	0.036
*Oscillibacter*	0.60 ± 0.22	0.00 ± 0.00	0.044				
*Eubacterium_fissicatena_group*	0.34 ± 0.03	0.00 ± 0.00	0.044				
*Lachnoclostridium*	0.32 ± 0.07	0.05 ± 0.03	0.044				
*Clostridium_sensu_stricto_1*	0.22 ± 0.14	6.69 ± 2.21	0.033				
*Staphylococcus*	0.21 ± 0.05	2.72 ± 0.32	0.000				
*Rothia*	0.18 ± 0.03	0.89 ± 0.20	0.015				
*Veillonella*	0.03 ± 0.01	2.25 ± 0.44	0.004				
*Moraxella*	0.01 ± 0.00	0.81 ± 0.26	0.026				

**TABLE 4:** The differential jejunal bacterial community of newborn piglets between the control and experimental groups within the same genotype (%).

	Hoxa1^–/–^ piglets			Non-Hoxa1^–/–^ piglets			
			
	Control group (KD-1)	Experimental group (KC-1)	*p*-value		Control group (KD-2)	Experimental group (KC-2)	*p*-value
**Phylum level**				**Genus level**			
Firmicutes	28.78 ± 7.11	55.36 ± 8.11	0.034	*Rothia*	0.89 ± 0.02	0.14 ± 0.03	0.019
Patescibacteria	1.63 ± 0.62	0.04 ± 0.00	0.049	*Moraxella*	0.81 ± 0.26	0.03 ± 0.00	0.028
Genus level							
*Eubacterium_fissicatena_group*	0.34 ± 0.03	0.00 ± 0.00	0.046				

Data in Table 4 show that Hoxa1^–/–^ neonatal piglets from the experimental group had significantly higher relative abundance of Firmicutes (*p* < 0.05) and lower relative abundance of Patescibacteria (*p* < 0.05) at the phylum level and lower relative abundance of *Eubacterium_fissicatena_group* (*p* < 0.05) at the genus level, respectively, in the jejunal meconium than Hoxa1^–/–^ neonatal piglets from the control group, and this means that maternal ATRA administration increased the relative abundance of phylum Firmicutes and decreased the relative abundances of phylum Patescibacteria and genus *Eubacterium_fissicatena_group* of Hoxa1^–/–^ neonatal piglets, respectively. Non-Hoxa1^–/–^ neonatal piglets from the experimental group only had significantly lower relative abundances of *Rothia* (*p* < 0.05) and *Moraxella* (*p* < 0.05) at the genus level in the jejunal meconium than non-Hoxa1^–/–^ neonatal piglets from the control group.

### Differential Abundance Analysis of Ileal Bacterial Community

The differential relative abundances of bacterial taxa of ileal meconium of neonatal piglets between different genotypes within the same treatment group are presented as percentages in Table 5. Three bacterial taxa with differential abundances at the phylum level and 12 bacterial taxa with differential abundances at the genus level were identified in the ileal meconium between Hoxa1^–/–^ and non-Hoxa1^–/–^ neonatal piglets from the control group (Table 5), and Hoxa1^–/–^ neonatal piglets had significantly lower relative abundances of Firmicutes (*p* < 0.01) at the phylum level and of *Lactobacillus* (*p* < 0.01) and *Moraxella* (*p* < 0.01) at the genus level in the ileal meconium, respectively, than non-Hoxa1^–/–^ neonatal piglets, but Hoxa1^–/–^ neonatal piglets had significantly higher relative abundances of Bacteroidetes (*p* < 0.05) and Deferribacteres (*p* < 0.05) at the phylum level and of *Prevotellaceae_UCG-001* (*p* < 0.05), *Ruminococcaceae_UCG-014* (*p* < 0.05), *Eubacterium_xylanophilum_group* (*p* < 0.05), *Ruminiclostridium* (*p* < 0.05), *Acinetobacter* (*p* < 0.05), *Ruminococcus_1* (*p* < 0.05), *Mucispirillum* (*p* < 0.05), *Ruminococcaceae_UCG-005* (*p* < 0.05), and *Eubacterium_coprostanoligenes_group* (*p* < 0.05) at the genus level in the ileal meconium, respectively, than non-Hoxa1^–/–^ neonatal piglets. After maternal ATRA administration, Hoxa1^–/–^ neonatal piglets only had significantly lower relative abundance of Spirochaetes at the phylum level in the ileal meconium than non-Hoxa1^–/–^ neonatal piglets (*p* < 0.05).

**TABLE 5:** The differential ileal bacterial community of newborn piglets between different genotypes within the same treatment group (%).

	Control group			Experimental group			
			
	Hoxa1^–/–^ piglets (HD-1)	Non-Hoxa1^–/–^ piglets (HD-2)	*p*-value		Hoxa1^–/–^ piglets (HC-1)	Non-Hoxa1^–/–^ piglets (HC-2)	*p*-value
**Phylum level**				**Phylum level**			
Firmicutes	27.60 ± 9.22	81.17 ± 3.79	0.001	Spirochaetes	0.00 ± 0.00	0.02 ± 0.00	0.027
Bacteroidetes	17.05 ± 4.99	1.32 ± 0.76	0.025				
Deferribacteres	0.11 ± 0.04	0.00 ± 0.00	0.037				
**Genus level**							
*Lactobacillus*	2.19 ± 0.39	57.75 ± 5.67	0.000				
*Prevotellaceae_UCG-001*	0.34 ± 0.12	0.01 ± 0.00	0.037				
*Ruminococcaceae_UCG-014*	0.33 ± 0.09	0.00 ± 0.00	0.017				
*Eubacterium_xylanophilum_group*	0.25 ± 0.09	0.00 ± 0.00	0.042				
*Ruminiclostridium*	0.23 ± 0.08	0.00 ± 0.00	0.039				
*Acinetobacter*	0.21 ± 0.06	0.04 ± 0.01	0.031				
*Veillonella*	0.17 ± 0.08	3.18 ± 1.08	0.038				
*Ruminococcus_1*	0.12 ± 0.04	0.00 ± 0.00	0.034				
*Mucispirillum*	0.11 ± 0.04	0.00 ± 0.00	0.037				
*Ruminococcaceae_UCG-005*	0.09 ± 0.03	0.00 ± 0.00	0.034				
*Eubacterium_coprostanoligenes_group*	0.09 ± 0.03	0.00 ± 0.00	0.040				
*Moraxella*	0.00 ± 0.00	0.70 ± 0.05	0.002				

The differential relative abundances of bacterial taxa of ileal meconium of neonatal piglets with the same genotype between different treatment groups are presented as percentages in Table 6. Data indicated that Hoxa1^–/–^ piglets from the experimental group had significantly lower relative abundances of Deferribacteres (*p* < 0.05) at the phylum level and of *Mucispirillum* (*p* < 0.05) and *Ruminococcaceae_UCG-005* (*p* < 0.05) at the genus level in the ileal meconium, respectively, than Hoxa1^–/–^ neonatal piglets from the control group, and non-Hoxa1^–/–^ neonatal piglets from the experimental group had significantly lower relative abundances of Firmicutes (*p* < 0.05) and Actinobacteria (*p* < 0.05) at the phylum level and of *Lactobacillus* (*p* < 0.01), *Staphylococcus* (*p* < 0.05), *Moraxella* (*p* < 0.01), *Rothia* (*p* < 0.01), and *Pedobacter* (*p* < 0.05) at the genus level in the ileal meconium, respectively, than non-Hoxa1^–/–^ neonatal piglets from the control group.

**TABLE 6:** The differential ileal bacterial community of newborn piglets with the same genotype between the control and experimental groups (%).

	Hoxa1^–/–^ piglets				Non-Hoxa1^–/–^ piglets		
			
	Control group (HD-1)	Experimental group (HC-1)	*p*-value		Control group (HD-2)	Experimental group (HC-2)	*p*-value
**Phylum level**				**Phylum level**			
Deferribacteres	0.11 ± 0.04	0.00 ± 0.00	0.038	Firmicutes	81.17 ± 3.79	52.16 ± 6.18	0.035
				Actinobacteria	1.76 ± 0.62	0.53 ± 0.19	0.039
**Genus level**				**Genus level**			
*Mucispirillum*	0.11 ± 0.04	0.00 ± 0.00	0.038	*Lactobacillus*	57.75 ± 5.67	16.78 ± 7.48	0.002
*Ruminococcaceae_UCG-005*	0.09 ± 0.03	0.00 ± 0.00	0.036	*Staphylococcus*	1.35 ± 0.25	0.20 ± 0.07	0.022
				*Moraxella*	0.70 ± 0.05	0.06 ± 0.01	0.001
				*Rothia*	0.66 ± 0.09	0.05 ± 0.02	0.001
				*Pedobacter*	0.06 ± 0.01	0.01 ± 0.00	0.036

### Predicted Gene Functions of Bacteria

**FIGURE 3:**
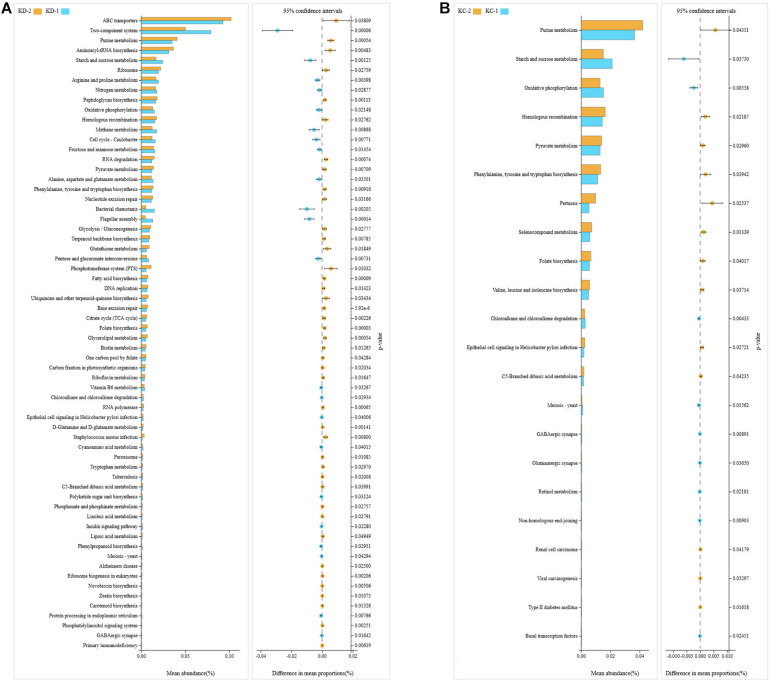
KEGG pathways for bacteria in jejunal meconium of neonatal piglets before suckling. KD-1: bacteria of jejunal meconium of Hoxa1^− /−^ neonatal piglets from the control group. KD-2: bacteria of jejunal meconium of non-Hoxa1^− /−^ neonatal piglets from the control group. KC-1: bacteria of jejunal meconium of Hoxa1^− /−^ neonatal piglets from the experimental group. KC-2: bacteria of jejunal meconium of non-Hoxa1^− /−^ neonatal piglets from the experimental group. **(A)** Control group. **(B)** Experimental group.

**FIGURE 4:**
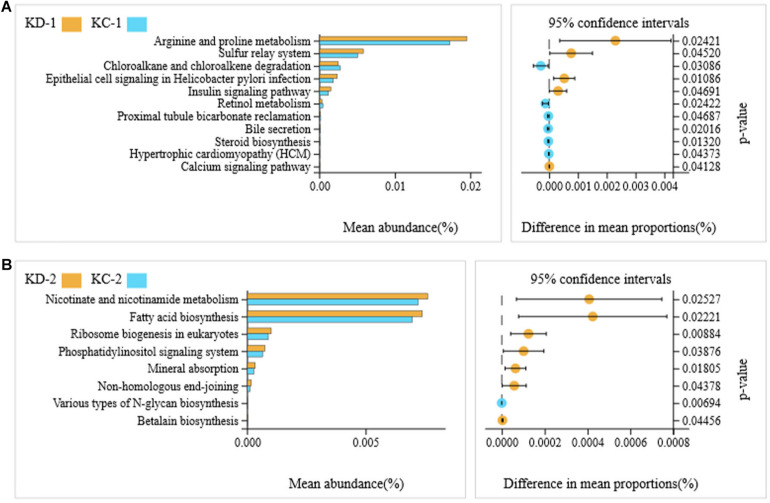
KEGG pathways for bacteria in jejunal meconium of neonatal piglets before suckling. KD-1: bacteria of jejunal meconium of Hoxa1^− /−^ neonatal piglets from the control group. KC-1: bacteria of jejunal meconium of Hoxa1^− /−^ neonatal piglets from the experimental group. KD-2: bacteria of jejunal meconium of non-Hoxa1^− /−^ neonatal piglets from the control group. KC-2: bacteria of jejunal meconium of non-Hoxa1^− /−^ neonatal piglets from the experimental group. **(A)** Hoxa1^–/–^ piglets. **(B)** Non-Hoxa1^–/–^piglets.

The Tax4Fun package in R software was used to predict the functional potentials of intestinal bacteria based on the KEGG KO terms at level 3 with the observed 16S rRNA gene sequences, and the Welch’s *t*-test results indicated that there were significant differences in 64 microbial metabolic pathways between jejunal bacteria of non-Hoxa1^–/–^ (KD-2) and Hoxa1^–/–^ (KD-1) neonatal piglets from the control group. The abundances of 22 functions were significantly higher and the abundances of 42 functions were significantly lower in KD-1 compared with KD-2 (Figure 3A). After maternal ATRA administration, a total of 22 microbial metabolic pathways were significantly different between jejunal bacteria of non-Hoxa1^–/–^ (KC-2) and Hoxa1^–/–^ (KC-1) neonatal piglets from the experimental group, and KC-1 had significantly higher expression of genes involved in starch and sucrose metabolism, oxidative phosphorylation, chloroalkane and chloroalkene degradation, meiosis, GABAergic synapse, glutamatergic synapse, retinol metabolism, non-homologous end-joining, and basal transcription factors compared with KC-2 (Figure 3B). KC-1 had significantly higher expression of genes involved in chloroalkane and chloroalkene degradation, retinol metabolism, proximal tubule bicarbonate reclamation, bile secretion, steroid biosynthesis, and hypertrophic cardiomyopathy than KD-1 (Figure 4A), and KC-2 had significantly lower expression of genes involved in nicotinate and nicotinamide metabolism, fatty acid biosynthesis, ribosome biogenesis in eukaryotes, phosphatidylinositol signaling system, mineral absorption, non-homologous end-joining, and betalain biosynthesis than KD-2 (Figure 4B).

**FIGURE 5:**
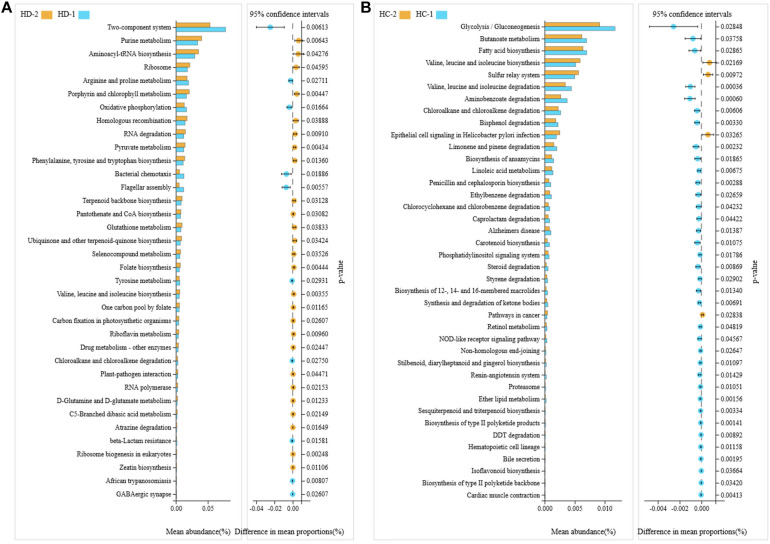
KEGG pathways for bacteria in ileal meconium of neonatal piglets before suckling. HD-1: bacteria of ileal meconium of Hoxa1^− /−^ neonatal piglets from the control group. HD-2: bacteria of ileal meconium of non-Hoxa1^− /−^ neonatal piglets from the control group. HC−1: bacteria of ileal meconium of Hoxa1^− /−^ neonatal piglets from the experimental group. HC-2: bacteria of ileal meconium of non-Hoxa1^− /−^ neonatal piglets from the experimental group. **(A)** Control group. **(B)** Experimental group.

**FIGURE 6:**
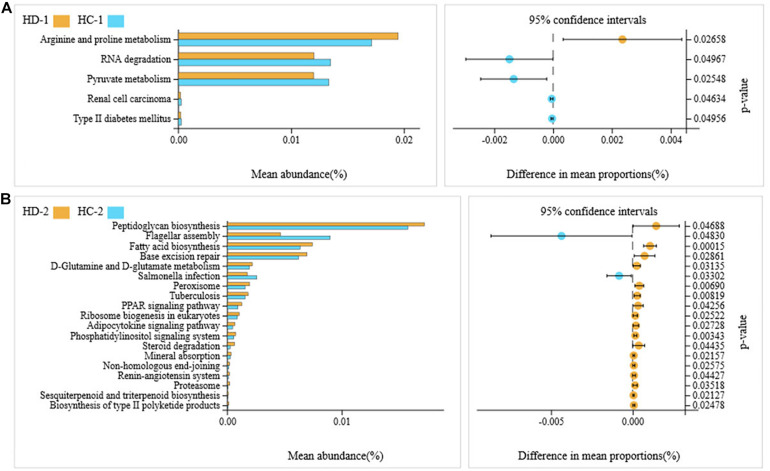
KEGG pathways for bacteria in ileal meconium of neonatal piglets before suckling. HD-1: bacteria of ileal meconium of Hoxa1^− /−^ neonatal piglets from the control group. HC-1: bacteria of ileal meconium of Hoxa1^− /−^ neonatal piglets from the experimental group. HD-2: bacteria of ileal meconium of non-Hoxa1^− /−^ neonatal piglets from the control group. HC-2: bacteria of ileal meconium of non-Hoxa1^− /−^ neonatal piglets from the experimental group. **(A)** Hoxa1^–/–^ piglets. **(B)** Non-Hoxa1^–/–^piglets.

A total of 36 microbial metabolic pathways were significantly different between ileal bacteria of non-Hoxa1^–/–^ (HD-2) and Hoxa1^–/–^ (HD-1) neonatal piglets from the control group, and 10 pathways were significantly upregulated and 26 pathways were significantly downregulated in HD-1 compared with HD-2 (Figure 5A). After maternal ATRA administration, 40 microbial metabolic pathways were significantly different between ileal bacteria of non-Hoxa1^–/–^ (HC-2) and Hoxa1^–/–^ (HC-1) neonatal piglets from the experimental group, and 36 pathways were significantly upregulated and 4 pathways were significantly downregulated in HC-1 compared with HC-2 (Figure 5B). HC-1 had significantly lower expression of genes involved in arginine and proline metabolism and significantly higher expression of genes involved in RNA degradation, pyruvate metabolism, renal cell carcinoma, and type II diabetes mellitus than HD-1 (Figure 6A), and HC-2 had significantly higher expression of genes involved in flagellar assembly and *Salmonella* infection and significantly lower expression of genes involved in peptidoglycan biosynthesis, fatty acid biosynthesis, base excision repair, D-glutamine and D-glutamate metabolism, peroxisome, tuberculosis, peroxisome proliferator-activated receptors (PPAR) signaling pathway, ribosome biogenesis in eukaryotes, adipocytokine signaling pathway, phosphatidylinositol signaling system, steroid degradation, mineral absorption, non-homologous end-joining, renin–angiotensin system, proteasome, sesquiterpenoid and triterpenoid biosynthesis, and biosynthesis of type II polyketide products than HD-2 (Figure 6B).

## Discussion

The normal colonization and development of intestinal microbiota is crucial for the normal function of the physiology and immunity of the host (Chung et al., 2012; Furusawa et al., 2013). The highly diverse intestine microbiota is generally considered beneficial for host health and is also regarded as a sign of mature intestine microbiota (Turnbaugh et al., 2007; Le Chatelier et al., 2013), but some studies found that premature development and diversification of the gut microbiota may negatively impact immune function and the highly diverse and rich bacterial community is probably not beneficial for the immature intestinal tract of young animals (Nylund et al., 2013; Wood et al., 2015). The data in our study also indicated that decreasing the number and alpha diversity of small intestinal bacterial community is beneficial to the neonatal piglets, because Hoxa1^–/–^ neonatal piglets delivered by sows with ATRA administration during pregnancy had lower number and alpha diversity of small intestinal bacterial community, heavier birth live weight, and less symptom with dyspnea than Hoxa1^–/–^ neonatal piglets born by sows with no ATRA administration.

Maternal genetics (Goodrich et al., 2014), gene mutation (Strati et al., 2016), delivery methods (Bi et al., 2021; Husso et al., 2021), age (Yatsunenko et al., 2012), disease (Alkanani et al., 2015), diet (David et al., 2014; Angoa-Pérez et al., 2020), and medication (Forslund et al., 2013) are the dominant factors in shaping the composition and abundance of intestinal microbiota. It is reported that the composition of intestinal bacterial communities in human and other mammals after suckling is mainly dominated by the phyla Firmicutes and Bacteroidetes, followed by the phyla Proteobacteria, Actinobacteria, and Verrucomicrobia (Gill et al., 2006; Duncan et al., 2008; Ley et al., 2008); however, the compositions of bacteria in the meconium of fetal bovine and lamb delivered by cesarean section were primarily composed of the phyla Proteobacteria and Firmicutes instead of Firmicutes and Bacteroidetes (Bi et al., 2021; Husso et al., 2021), and data in our study also indicated that Firmicutes and Proteobacteria were the most abundant phyla in the meconium of vaginal-delivered neonatal piglets before suckling.

Gene mutation can cause dysbiosis of gut microbiota by altering the relative abundance of gut microbiota and often develop a wide variety of diseases (Johansson et al., 2011; Alkanani et al., 2015; Strati et al., 2016). Microbial dysbiosis of the gut can destroy body immunity and intestinal mucosal barrier and increase gut permeability *via* the outgrowth of pathogens or the imbalance production of chemical substances in the gut (Earley et al., 2015). Mutations in the *MECP2* gene decreased the relative abundances of Firmicutes and Bacteroidetes and increased the relative abundance of Actinobacteria at the phyla level of patients compared with healthy controls (Strati et al., 2016). Our study showed that Hoxa1 mutation changed the relative abundances of small intestinal microbiota, Hoxa1^–/–^ neonatal piglets had lower relative abundances of phylum Firmicutes and genera *Lactobacillus*, *Clostridium_sensu_stricto_1*, *Rothia*, and *Veillonella* and higher relative abundances of phyla Proteobacteria, Bacteroidetes, and Verrucomicrobia and genera *Escherichia–Shigella* and *Akkermansia* than non-Hoxa1^–/–^ neonate piglets.

The normal ratio of Firmicutes to Bacteroidetes and the relative abundance of microbiota are essential for host health, and gene mutation often changes the Firmicutes to Bacteroidetes ratio and the microbial relative abundance. Tougaard et al. (2015) reported that tumor necrosis factor-like ligand 1A gene knockout mice had lower Firmicutes/Bacteroidetes ratio in the cecum content compared with wild-type mice (Tougaard et al., 2015), and the results of this study also indicated that Hoxa1 mutation resulted in a significantly lower ratio of Firmicutes to Bacteroidetes in the small intestine of Hoxa1^–/–^ neonatal piglets than that of non-Hoxa1^–/–^ neonatal piglets. Alteration in the ratio of Firmicutes to Bacteroidetes and the relative abundances of microbiota often develop into different kinds of diseases in the host. The increased ratio of Firmicutes to Bacteroidetes was reported in obese animals (Bäckhed et al., 2004), and the decreased ratio between Firmicutes and Bacteroidetes was associated with a higher risk of developing type 1 diabetes or celiac disease (Murri et al., 2013; Calderón de la Barca et al., 2020) or autism (Finegold et al., 2010) in children. The combination of significantly decreased ratio of Firmicutes to Bacteroidetes; the lower relative abundances of Firmicutes, *Lactobacillus*, *Clostridium_sensu_stricto_1*, *Staphylococcus*, *Rothia*, and *Veillonella*; and the higher relative abundances of Bacteroidetes and Patescibacteria might be some of the important factors to develop in Hoxa1^–/–^ neonatal piglets undesirable symptoms of bad birth live weight, dyspnea, and death. Previous studies reported that the significantly decreased relative abundances of phylum Firmicutes and of genus *Lactobacillus* were correlated with prenatal stress (Zijlmans et al., 2015), multiple sclerosis (Chen et al., 2016), type 1 diabetes (Murri et al., 2013; Alkanani et al., 2015), and diarrheal disease (Zhuang et al., 2017). The decreased levels of *Veillonella*, *Lachnospira*, *Rothia*, *Roseburia*, and *Faecalibacterium* were also associated with asthma in children (Hilty et al., 2010; Hufnagl et al., 2020). Decreasing the numbers of Firmicutes, *Lactobacillus*, *Veillonella*, *Rothia*, and *Clostridium_sensu_stricto_1* will increase the growth and colonization of bacterial pathogens (Kim et al., 2017; Vitetta et al., 2017) and weaken gastrointestinal digestion, development, and immune functions (Edwards et al., 2017; Granja-Salcedo et al., 2017). The increased relative abundance of Bacteroidetes can generate more propionate, and the surplus of propionate can lower food intake, increase energy expenditures (Chambers et al., 2015, 2018), and facilitate the absorption of iron (Sfera et al., 2020); chronic iron overload increases the risk of reactive oxygen species and DNA damage (Sfera et al., 2020) and decreases weight gain (Calarge et al., 2016).

Preventing microbial dysbiosis in early life can reduce the risk of diseases such as intrauterine growth retardation (IUGR), allergic asthma, type 1 diabetes, and diarrhea. Studies showed that increased *Lactobacillus* can modulate gut microbiota dysbiosis (Wu et al., 2019), and early colonization with *Lactobacillus* during the infant period can reduce the risk of allergic asthma (Johansson et al., 2011). The data of our studies demonstrated that maternal ATRA administration increased the birth live weight of Hoxa1^–/–^ neonatal piglets (Zhou et al., 2021). The increased ratio of Firmicutes to Bacteroidetes; the higher relative abundances of Firmicutes, Proteobacteria, and *Lactobacillus*; and the lower relative abundance of Bacteroidetes were observed in the meconium of the small intestine of Hoxa1^–/–^ neonatal piglets, and the symptom of respiratory distress was not observed in Hoxa1^–/–^ neonatal piglets delivered by sows with ATRA administration.

Gut microbiota may play important roles in the health and nutrient metabolism of the host, and prenatal colonization of a metabolically active microbiome is clinically vital for the health development of the fetus (Bi et al., 2021). A previous study found that microbiota in the gut of fetal lambs had high enrichment of KEGG pathways related to carbohydrate metabolism, energy metabolism, signal transduction, and amino acid metabolism (Bi et al., 2021). Our study indicated that meconium bacteria of the small intestine of neonatal piglets have functional enrichments mainly in membrane transport (ABC transporters), signal transduction (two-component system), nucleotide metabolism (purine metabolism), translation (aminoacyl-tRNA biosynthesis, ribosome), carbohydrate metabolism (starch and sucrose metabolism, fructose and mannose metabolism, pyruvate metabolism), amino acid metabolism (arginine and proline metabolism; alanine, aspartate, and glutamate metabolism; phenylalanine, tyrosine, and tryptophan biosynthesis), energy metabolism (nitrogen metabolism, oxidative phosphorylation, methane metabolism), and cell motility (bacterial chemotaxis). Hoxa1^–/–^ neonatal piglets had lower functional enrichments of small intestinal bacteria in ABC transporters, purine metabolism, aminoacyl-tRNA biosynthesis, and ribosome and pyruvate metabolism and higher functional enrichments of small intestinal bacteria in two-component system; starch and sucrose metabolism; fructose and mannose metabolism; arginine and proline metabolism; alanine, aspartate, and glutamate metabolism; nitrogen metabolism; and bacterial chemotaxis than non-Hoxa1^–/–^ neonatal piglets. In addition, maternal ATRA administration during pregnancy not only can shape the functional enrichments of small intestinal bacteria of Hoxa1^–/–^ neonatal piglets but also can alter the functional enrichments of small intestinal bacteria of non-Hoxa1^–/–^ neonatal piglets. Data showed that non-Hoxa1^–/–^ neonatal piglets delivered by sows with ATRA administration had significantly lower functional enrichments of small intestinal bacteria in nicotinate and nicotinamide metabolism, fatty acid biosynthesis, ribosome biogenesis in eukaryotes, phosphatidylinositol signaling system, mineral absorption, peptidoglycan biosynthesis, base excision repair, D-glutamine and D-glutamate metabolism, tuberculosis, and PPAR signaling pathway and significantly higher functional enrichments of small intestinal bacteria in flagellar assembly and *Salmonella* infection than non-Hoxa1^–/–^ neonatal piglets born by sows with no ATRA administration.

## Conclusion

Hoxa1 mutation altered the diversity of the bacterial community and the relative abundances of several dominant taxa; Hoxa1^–/–^ neonatal piglets had significantly higher alpha diversity of bacterial community, lower phylum Firmicutes and genus *Lactobacillus*, and higher phylum Bacteroidetes than non-Hoxa1^–/–^ neonatal piglets in the meconium of the jejunum and ileum. Maternal ATRA administration altered the bacterial diversity and the relative abundances of dominant taxa of Hoxa1^–/–^ and non-Hoxa1^–/–^ neonatal piglets; Hoxa1^–/–^ neonatal piglets delivered by sows with ATRA administration had lower alpha diversity of bacterial community and significantly higher relative abundance of phylum Firmicutes than Hoxa1^–/–^ neonatal piglets born by sows with no ATRA administration, but had higher alpha diversity of bacterial community, higher relative abundances of phyla Proteobacteria and Bacteroidetes, and lower relative abundances of phylum Firmicutes and genus *Lactobacillus* than non-Hoxa1^–/–^ neonatal piglets delivered by sows with ATRA administration. Compared with non-Honx1^–/–^ neonatal piglets delivered by sows with no ATRA administration, non-Hoxa1^–/–^ neonatal piglets born by sows with ATRA administration had higher alpha diversity of bacterial community and lower relative abundances of the genera *Rothia* and *Moraxella*.

## Data Availability Statement

The datasets presented in this study can be found in online repositories. The names of the repository/repositories and accession number(s) can be found in the article/supplementary material.

## Ethics Statement

The animal study was reviewed and approved by the Ethics Committee for Animal Experimentation of Jiangxi Agricultural University. Written informed consent was obtained from the owners for the participation of their animals in this study.

## Author Contributions

YH conceived the study. YH and WL designed the study. HZ, HW, YC, and WZ performed the experiments. HZ performed the data analysis. YH and HZ writing the manuscript. All authors read and approved the final manuscript.

## Conflict of Interest

The authors declare that the research was conducted in the absence of any commercial or financial relationships that could be construed as a potential conflict of interest.

## Publisher’s Note

All claims expressed in this article are solely those of the authors and do not necessarily represent those of their affiliated organizations, or those of the publisher, the editors and the reviewers. Any product that may be evaluated in this article, or claim that may be made by its manufacturer, is not guaranteed or endorsed by the publisher.
